# Birth size, risk factors across life and cognition in late life: protocol of prospective longitudinal follow-up of the MYNAH (MYsore studies of Natal effects on Ageing and Health) cohort

**DOI:** 10.1136/bmjopen-2016-012552

**Published:** 2017-02-16

**Authors:** Murali Krishna, G Mohan Kumar, S R Veena, G V Krishnaveni, Kalyanaraman Kumaran, Samuel Christaprasad Karat, Patsy Coakley, Clive Osmond, John R M Copeland, Giriraj Chandak, Dattatray Bhat, Mathew Varghese, Martin Prince, Caroline Fall

**Affiliations:** 1 Epidemiology Research Unit, CSI Holdsworth Memorial Hospital, Mysore, Karnataka, India; 2 MRC Lifecourse Epidemiology Unit, University of Southampton, Southampton General Hospital, Southampton, UK; 3 Department of Psychiatry, University of Liverpool, Liverpool, UK; 4 Centre for Cellular and Molecular Biology, Hyderabad, Telangana, India; 5 Diabetic Unit, KEM Hospital, Pune, Maharashtra, India; 6 Department of Psychiatry, National Institute of Mental Health and Neurosciences, Bangalore, Karnataka, India; 7 Institute of Psychiatry, De Crespigny Park, Kings College, London, UK

**Keywords:** Cognition, Late life, programming, birth weight, fetal growth

## Abstract

**Introduction:**

For late-life neurocognitive disorders, as for other late-life chronic diseases, much recent interest has focused on the possible relevance of Developmental Origins of Health and Disease (DOHaD). Programming by undernutrition in utero, followed by overnutrition in adult life may lead to an increased risk, possibly mediated through cardiovascular and metabolic pathways. This study will specifically examine, if lower birth weight is associated with poorer cognitive functioning in late life in a south Indian population.

**Methods and analysis:**

From 1934 onwards, the birth weight, length and head circumference of all babies born in the CSI Holdsworth Memorial Hospital, Mysore, India, were recorded in obstetric notes. Approximately 800 men and women from the Mysore Birth Records Cohort aged above 55 years, and a reliable informant for each, will be asked to participate in a single cross-sectional baseline assessment for cognitive function, mental health and cardiometabolic disorders. Participants will be assessed for hypertension, type-2 diabetes and coronary heart disease, nutritional status, health behaviours and lifestyles, family living arrangements, economic status, social support and social networks. Additional investigations include blood tests (for diabetes, insulin resistance, dyslipidaemia, anaemia, vitamin B_12_ and folate deficiency, hyperhomocysteinemia, renal impairment, thyroid disease and Apolipoprotein E genotype), anthropometry, ECG, blood pressure, spirometry and body composition (bioimpedance). We will develop an analysis plan, first using traditional univariate and multivariable analytical paradigms with independent, dependent and mediating/confounding/interacting variables to test the main hypotheses.

**Ethics and dissemination:**

This study has been approved by the research ethics committee of CSI Holdsworth Memorial Hospital. The findings will be disseminated locally and at international meetings, and will be published in open access peer reviewed journals.

**Strengths and limitations of this study**

- This study is the first lifecourse birth records cohort study in the developing world with cognitive, physical health and mental health outcomes in older adults.
- This will add to the scientific understanding of the early life origins of disease, and mediating effect of cardiometabolic disorders in adult and late life on late life cognitive abilities and mental health in an Indian population.
- There have been large losses to follow-up since birth, especially between birth and the first adult follow-up, and the implications of these losses have to be thought through in interpreting the results of this study.

## Background

Neurocognitive disorders are a major cause of disability and mortality in late life and are associated with high costs for health systems and society.^1^ It is estimated that in India, 3.7 million people aged over 60 have dementia and this is expected to increase twofold by 2030 and threefold by 2050 because of steady growth in the older population and stable increments in life expectancy.^2^ In the accompanying health transition, non-communicable diseases have assumed a progressively greater significance in low and middle-income countries (LMICs) like India. Although neurocognitive disorders are the second highest source of burden after tropical diseases, research in India is minimal.^1^ ^2^

Recent research suggests that the quality of nutrition during intrauterine development, reflected crudely in size at birth, is an important determinant of lifelong function, health and disease risk.^3^ Birth weight and head circumference at birth are indicators of intrauterine growth and brain development, respectively.^4^ Larger birth weight, the most widely researched birth size measure, is associated with better cognitive function from infancy through the third decade of life in several populations and countries, independent of social background.^5^ However, this association between birth weight and cognition diminishes as individuals reach middle age.^6^ Cohort studies with follow-up through late life have been sparse, making the size of effects in late life unclear.^6^ ^7^ Head circumference at birth has shown a similar pattern to birth weight, such that associations observed in young cohorts were not replicated in the older adults in follow-up studies.^8^ Conditions in early postnatal life and childhood may supersede and compensate for fetal vulnerabilities such as small birth size and moderate long-term cognitive abilities.

Adult cardiometabolic disorders are also thought to have origins in fetal life.^8^ Low birth weight is linked to adult cardiometabolic disorders (type-2 diabetes, coronary heart disease, hypertension and metabolic syndrome).^9–12^ These disorders are independently associated with impaired cognition and dementia in old age.^13^ While hypertension, obesity and hyperlipidaemia in midlife predict an increased risk of later dementia, associations become inverted in late life.^14–16^ Understanding long-term trajectories in Body Mass Index (BMI), blood pressure and cholesterol among those who do and do not develop cognitive impairment can be clarified by conducting prospective lifecourse epidemiological studies.

Much interest has focused on the possible relevance of Barker's fetal and developmental origins of adult disease (DOHaD) hypothesis with two plausible pathways to late-onset cognitive impairment and dementia.^17^ ^18^

1. a direct effect of reduced intrauterine nutrition (reflected in smaller birth size) on fetal brain development and leading to decreased cognitive abilities and hence reduced cognitive reserve;^17^
2. programming of metabolism by undernutrition in very early life, exacerbated by overnutrition in adult life, leading to increased risk mediated through cardiometabolic disease.^13^ ^17^ ^18^

Cognition in late life is impacted by the cumulative effect of nutrition, education, social and family environment in early life and midlife.^17^ ^18^ Adverse lifetime socioeconomic conditions, including lower educational attainment, less complex occupational activities, area of birth and childhood residence are associated with cognitive impairment and dementia.^17^ ^18^ However, only a few studies have examined the role of mediating factors between socioeconomic deprivation across the life course and cognitive ageing.^17^ ^19^ Data spanning the lifecourse into old age are hard to come by in LMICs. The Mysore Birth Records Cohort^20^ in south India represents a unique resource for the study of the lifecourse aetiology of cognitive ageing in a radically different cultural context from those where such studies have previously been conducted.

### Mysore Birth Records Cohort

CSI Holdsworth Memorial Hospital (HMH), Mysore, India has preserved obstetric records since 1934 until the present date. These include the birth weight, length and head circumference of all babies born in the hospital, as well as maternal weight and pelvic diameters. During 1993–2001, in a collaborative study with Barker's group at the MRC Lifecourse Epidemiology Unit, University of Southampton, UK, the records were used to trace people born in HMH between 1934 and 1966. The tracing process resulted in 3427 men and women being matched to their birth records.^20^

Of those born between 1934 and 1966, 1069 participated in a study between 1993 and 1999 to evaluate the relationship between size at birth and adult cardiometabolic disorders.^21–27^ A subset (n=435) was assessed for cardiac dimensions and arterial compliance 3–4 years later.^25^ ^26^ Ten years after the first study, 383 participated in a follow-up study for incident diabetes and metabolic syndrome between 2003 and 2004.^27^

The 3427 cohort members who were retraced and matched to their birth records in 1993–2001 were only a small percentage (14%) of all the births in HMH during 1934–1966, and of these ∼30% (n=1069) took part in the previous studies of the cohort. Compared with the remainder of the original births, the 3427 individuals who were traced were 48 g heavier (95% CI 28 to 68, p<0.001), 0.25 cm longer (95% CI 0.08 to 0.42, p=0.005) and had a 0.092 cm larger head circumference (95% CI −0.004 to 0.188; p=0.06) at birth. Compared with the 3427, the 1069 men and women who were studied were slightly lighter and shorter, but these differences were not statistically significant (birth weight −36 g (95% CI −74 to 2, p=0.07); birth length −0.26 cm (95% CI −0.55 to 0.03, p=0.08).^20^

Previous studies from the Mysore Birth Records Cohort provided partial support for the Barker hypothesis, with predicted associations between lower birth weight and coronary heart disease,^21^ insulin resistance^22^ and lower lung function.^24^ The prevalence and incidence of type-2 diabetes was higher compared with the west and was associated with short birth length rather than low birth weight.^21^ ^27^ Associations between cortisol concentration and cardiovascular risk factors were stronger.^23^ No associations were identified between birth weight and hypertension, left ventricular mass and arterial compliance.^25^ ^26^

For the purpose of this study, we aim to investigate ∼800 men and women from the Mysore Birth Records Cohort, born before 1959. There was no contact with the cohort between 2005 and 2011. Between 2011 and 2015 we retraced the cohort members and of the 1069, 186 are deceased, 727 are alive and 156 could not be traced. Recruitment for the study has started and the study is expected to be completed by 2017.

### Aims and objectives

The full potential of this cohort will be realised through repeated measures of cognitive and other functions in older age, to track cognitive trajectories, and the development of frailty across other physiological and organ systems. The purpose of this study is to establish baseline cognitive function in later life. We will be conducting cognitive assessments for all participants once during this study. However, to begin to explore pathways from early life growth and development to late-life impairment and decline, these assessments will be repeated in future follow-up studies of the cohort.

### Primary goal

This study aims to test the hypothesis that lower birth weight and smaller head circumference at birth are associated with poorer scores in tests of cognitive function in people aged 55 years and over, relative to age and education norms for the south Indian population. The mediating effects of childhood growth (proxied by adult head circumference and leg length), and cardiometabolic risk factors (in adult and late life) will be explored. Potential interactions will also be explored; for example, the association between birth weight and late-life cognitive ability may be modified by changes in socioeconomic status in midlife.

### Other key goals

1. To estimate the prevalence of cognitive impairment and its association with age, gender, education and socioeconomic position. We will investigate risk factors for cognitive impairment and test the following specific hypotheses:
  - Cardiovascular risk factors (diabetes, hypertension, large waist circumference, high waist:hip ratio, hypercholesterolaemia, hypertriglyceridaemia, metabolic syndrome, insulin resistance and smoking), coronary heart disease and stroke are associated with an increased risk of cognitive impairment and dementias, after controlling for confounding effects. Associations will be quantified as relative risks and population attributable fractions.
  - Micronutrient deficiencies (vitamin B_12_ and folate), and anaemia are associated with an increased risk of cognitive impairment.
  - ApoE4 (Apolipoprotein E4) genotype is associated with an increased risk of cognitive impairment and dementia.
  - Markers of socioeconomic adversity across life are associated with an increased risk of cognitive impairment and dementia.
2. We will study the social aetiology of late-life depression cross-sectionally including associations with relative and absolute poverty, ill health and disability, nutritional status, social support, life events, marital circumstances and living arrangements.

## Methods and analysis

### Study design

Approximately 800 men and women from the Mysore Birth Records Cohort, and the original 3427 who were traced and matched to their birth records, aged above 55 years, and a key informant will be asked to participate in a cross-sectional comprehensive 1-phase baseline assessment lasting ∼2–3 hours. Recruitment is expected to complete by 2017 and our analyses by 2018.

### Interviews and measures

A battery of cognitive tests and instruments developed, validated and normed in India by the 10/66 Dementia Research Group, suitable for people with little or no education, is administered in local languages.^28^ ^29^ ^30^ Interviews for participants and key informant are carried out in participants' own homes. Physical assessments and investigations are carried out the following day at the research unit. There is good evidence to suggest that adult head circumference and leg length are proxy markers for early life growth and development. We are measuring head circumference and leg length using standardised protocols in the current study. Table 1 lists the assessments and investigations that are conducted in this study.

**Table 1:** Instruments, assessments and investigations in the study protocol

Battery of Cognitive tests	The Community Screening Instrument for Dementia (CSI‘D’) COGSCORE incorporating the CERAD animal naming verbal fluency task. (CERAD—Consortium to Establish a register for Alzheimer's Disease)^37^ The modified CERAD 10-word list learning task with delayed recall^38^ Informant interview, the CSI‘D’ RELSCORE^37^, for evidence of cognitive and functional decline*
Instruments for diagnosis of dementia	Battery of cognitive tests (listed above) A structured clinical mental state interview, the Geriatric Mental State, which applies a computer algorithm^35^ An extended informant interview, the history and aetiology schedule—dementia diagnosis and subtype^39^* The NEUROEX, a brief fully structured neurological assessment^40^ Behavioural and psychological symptoms: assessed by Neuropsychiatric Inventory^41^*
Health status and physical health assessment	Self-reported global health by a structured interview†^28^ Self-reported diagnoses and treatments for these conditions†^28^ A self-reported list of 12 commonly occurring physical impairments†^42^ Activity limitation and participation restriction measured by the WHO—Disability Assessment Schedule II†^43^ Rose Angina Questionnaire^32^ Direct physical assessments: pulse rate, systolic and diastolic resting blood pressure, weight, height, leg length, head circumference, waist circumference, waist:hip ratio, skin fold thickness (subscapular, triceps and abdominal), calf circumference, hand grip test, bio impedance measurements, 12-lead ECG for Minnesota coding and 5 m walking test Reproductive status (for women)—menarche, menopause, reproductive period and number of children
A structured interview†	Specific cognitive risk factors Lifestyle and cardiovascular risk factors
Socioeconomic assessments	Modified Kuppuswamy Scale^44^**†** Standard of Living Index^45^**†**
Blood tests and genetic assay	*Blood tests*: Haemoglobin, glucose tolerance test, lipid profile, albumin, total protein, thyroid function tests, vitamin B_12_, folate, insulin and creatinine*Genetic assay*: ApoE lipoprotein

*Instruments administered to informants only.

**†**Instruments administered to the informants only if the participants have communication difficulties arising from cognitive problems, severe mental illness, deafness or mutism.

ApoE, Apolipoprotein E.

*Blood tests and genetic assays*: These are conducted according to a standard protocol. Samples for biochemical analyses are sent to the Diabetes Unit, KEM Hospital, Pune, India, whose laboratory participates in the UK National External Quality Assessment Service for insulin assays. The DNA sample was sent to the Centre for Cellular and Molecular Biology, Hyderabad, India, where DNA is extracted for APOE genotyping.

The WHO criteria are employed to diagnose diabetes and hypertension. Metabolic syndrome is diagnosed using International Diabetes Federation criteria.^31^ Diagnosis of coronary heart disease is derived from an algorithm, drawing information from the Rose Chest Pain Questionnaire,^32^ Minnesota ECG coding^33^ and a clinical history of cardiac revascularisation procedures. Diagnosis of coronary heart disease is made, if there is typical angina on Rose Chest Pain Questionnaire, or a history of cardiac revascularisation procedures or the presence of Minnesota codes 1–1 or 1–2 for major Q waves on the ECG.

*Outcomes*: Cognitive functioning as a continuous measure is obtained by administering the 10/66 battery of cognitive tests.^28–30^ Cognitive impairment is defined as 1.5 SDs or more below the norm for the age and educational group for individual tests, and scoring below that threshold on two out of three memory tests (immediate and delayed recall and Community Screening Instrument for Dementia (CSI‘D’) memory subscale). Dementia is defined by a score above a cut-off point of predicted probability of Diagnostic Statistical Manual for mental disorders (DSM) IV Dementia Syndrome^34^ from the logistic regression equation of the 10/66 dementia diagnostic algorithm.^28^ Depression is diagnosed from the computerised Geriatric Mental State Examination algorithm (GMS-AGECAT).^35^

### Power calculation and data analysis

Using a test at the 5% significance level, our sample size of 800 will provide 80% power to detect an association of 0.099 SDs of a continuous outcome (cognitive score) per SD of a continuous exposure (eg, birth weight). We will first calculate the prevalence of cognitive impairment (outcome of interest) and its association with age and gender.

Cardiometabolic data will be collected objectively using standardised protocols in this study, and are available from previous two studies. We will use these data to understand long-term trajectories in BMI, blood pressure, and cholesterol among those with and without cognitive impairment. Indicators of socioeconomic status have been collected from the cohort members at the time of birth (using data from maternity records), and during adult life using standard methods when they participated in previous studies. During this study, we will conduct an assessment of current socioeconomic position. We will examine for the association between lifecourse socioeconomic position and cognitive function in late life in our analyses.

We will develop an analysis plan, first using traditional univariate and multivariable analytical paradigms with independent, dependent and mediating/confounding/interacting variables to test the main hypotheses. We will then extend these analyses using path analysis and structural equation modelling by use of MPlus statistical software package to explore lifecourse pathways and mediated effects in more detail, allowing for the assumed causal ordering of observed and latent variables (eg, cognitive reserve) across the lifecourse.^36^ At the end of the recruitment, we plan to compare participants in the current study with non-participants. We will examine for any differences in birth size, age, gender mix, height, BMI, educational attainment, socioeconomic position and cardiometabolic variables collected previously.

## Ethics and dissemination

Full informed consent is obtained from the participants. If the participant is illiterate, verbal consent is obtained, witnessed and signed by a relative. If individuals were unable to consent (due to severe cognitive problems), witnessed assent is obtained from their nearest/authorised relative. The findings from this study will be disseminated locally and at international meetings, and will be published in open access peer review journals.

## Discussion

India is experiencing a phenomenal increase in adult cardiometabolic disorders, a trend attributed to wide spread intrauterine and infant-growth retardation and recent economic transition leading to unhealthy diets and lifestyles in childhood and adult life. These disorders in adult life increase the risk of cognitive impairment in old age where comorbidity with chronic non-communicable disorders is common. Despite the growing interest in chronic diseases in the elderly, there are few detailed data available on their prevalence, impact and developmental origins in the developing world.

This project constitutes a unique opportunity to begin to chart the epidemiologic transition and its impact on older persons in India. In addition to cognitive impairment, this cross-sectional survey includes ascertainment of mental disorders and other chronic conditions in late life. As well as providing a rich baseline for studying the aetiology of cognitive decline in future follow-up studies, this will allow us to explore the relative, independent and interactive contributions of various chronic conditions to late-life cognitive functioning. We will carry out comparative analyses using the publicly accessible data archive from the 10/66 Dementia Research Group's cross-sectional population-based surveys including over 19 000 older people from 10 developing countries. There have been large losses to follow-up since birth, especially between birth and the first adult follow-up, and the implications of these losses have to be thought through in interpreting the results of this study.

Numbers of older people are growing rapidly in India and the extent to which nutrition in early life and cardiometabolic risk factors across the lifespan are related to late-life cognition is of substantial public health relevance. Understanding the nature of this relationship and identification of critical periods could strengthen the rationale for policies to improve maternal and child nutrition and to develop interventions to prevent or modify chronic non-communicable disorders. Effective dissemination of findings from this research will raise awareness, inform evidence-based policymaking and enhance service development for older people in India.
